# Bufavirus in Feces of Patients with Gastroenteritis, Finland

**DOI:** 10.3201/eid2006.131674

**Published:** 2014-06

**Authors:** Elina Väisänen, Inka Kuisma, Tung G. Phan, Eric Delwart, Maija Lappalainen, Eveliina Tarkka, Klaus Hedman, Maria Söderlund-Venermo

**Affiliations:** Faculty of Medicine, University of Helsinki, Finland (E. Väisänen, I. Kuisma, K. Hedman, M. Söderlund-Venermo);; Blood Systems Research Institute, San Francisco, California, USA (T.G. Phan, E. Delwart);; University of California, San Francisco (T.G. Phan, E. Delwart);; Helsinki University Central Hospital, Helsinki (M. Lappalainen, E. Tarkka, K. Hedman)

**Keywords:** Parvovirus, bufavirus, bocavirus, viruses, Primate protoparvovirus, diarrhea, quantitative PCR, gastroenteritis, Burkina Faso, Finland

**To the Editor:** For nearly 3 decades, human parvovirus B19 (B19V) was considered to be the only pathogenic parvovirus found in humans. Since 2005, several new human parvoviruses have been found, including human bocaviruses (HBoV1–4) and human parvovirus 4 (PARV4) (*1**–**5*), and during 2012, metagenomic analysis of fecal samples from children in Burkina Faso with acute diarrhea showed a highly divergent parvovirus, which was named bufavirus (BuV) (*6*). Its sequence in the coding region showed <31% similarity with known parvoviruses, the closest genera being *Protoparvovirus* and *Amdoparvovirus*. Subsequent studies, on the basis of PCR results, showed that 4% of fecal samples from Burkina Faso (n = 98) and 1.6% from Tunisia (n = 63) harbored either of 2 genotypes of this new virus, which belongs to the species *Primate protoparvovirus 1* of the genus *Protoparvovirus* (*6**,**7*; http://ictvonline.org).

To assess the occurrence of BuV in northern Europe, we analyzed 629 fecal samples from patients of all ages (median 51.5 years, range 0–99) in Finland who had gastroenteritis. To gain a more complete representation of BuV occurrence, we obtained samples retrospectively from routine diagnostics for bacterial and viral gastroenteritis-inducing pathogens (HUSLAB, Helsinki University Central Hospital Laboratory Division, Helsinki, Finland) and analyzed all samples available during the collection periods.

The samples originally sent to HUSLAB for bacterial diagnosis (bacterial cohort, n = 243) had been analyzed during October 2012–March 2013 for *Salmonella* spp*., Shigella* spp., *Campylobacter* spp*., Yersinia* spp*., Vibrio cholerae*, and *Escherichia coli* (subtypes enterohemorraghica, enteropatogena, enterotoxigenic and enteroagregativa) by using culture or PCR (*8*). In 81 (33.3%) of the samples, >1 bacterial pathogen was found. 

The samples originally sent for viral diagnosis (viral cohort, n = 386) had been tested in HUSLAB for norovirus during April–May, 2013 by using reverse transcription quantitative PCE (RT-qPCR)(HUSLAB in-house). Further diagnosis for rotavirus and adenovirus had been requested by physicians from 105 (27.2%) of 386 samples (Diarlex MB antigen detection assay, Orion Diagnostica, Espoo, Finland), and for astrovirus from 33 (8.6%) samples (RT-PCR, HUSLAB in-house). A viral pathogen was discovered in 141 (36.5%) samples; in 139, the pathogen was norovirus.

The samples had been sent from diverse locations within Finland, and thus were not from a few isolated outbreaks. No further information on patients and samples was available for either cohort, and not enough samples were left for retrospective analysis of additional pathogens. The Ethics Committee of the Hospital District of Helsinki and Uusimaa approved the study.

BuV DNA was detected by using a new real-time qPCR with the following primers and probe: BuV forward, 5 ′-ACAGTGTAGACAGTGGATTCAAACTT-3 ′; BuV reverse, 5 ′-GTTGTGGTTGGATTGTGGTTAGTTC-3 ′; BuV qPCR probe, 5 ′-FAM-CGGAAGAGATTTTGACAGTGCYTAGCAA-BHQ1–3 ′. The detailed qPCR protocol is shown in the Technical Appendix. The analytical sensitivity of the RT-qPCR assay was 5–10 copies per reaction.

Of the 629 fecal samples, 7 (1.1%) were positive for BuV DNA, of which 4 were from the bacterial cohort and 3 from the viral cohort. BuV DNA quantity was low in all samples, ranging from 1.9 × 10^3^ to 3.2 × 10^4^ copies per milliliter of fecal supernatant (Table). In contrast to the original discovery of the virus in children with diarrhea (*6*), all positive samples were from adults (median age 53 years, range 21–89 years). All BuV DNA–positive results were confirmed by repeated BuV qPCR, by amplifying and sequencing another area of the virus, or by both methods (Table): all sequenced amplicons were more similar to the BuV genotype 1 (Technical Appendix Figure) (*6*). Two of the BuV-positive samples were from the same patient, taken 4 days apart, and the latter sample also harbored norovirus. The additional 6 BuV-positive samples were negative for the other viral or bacterial pathogens tested.

**Table:** Samples collected for bacterial and viral testing that were subsequently positive for bufavirus DNA*

No./Pt ID	Sample cohort	Quantity (copies/mL supernatant)	Age/y, sex	Pathogens tested for by HUSLAB†	Other pathogens found	Sampling date	Sequenced region, nt, divergence (%) from JX027295‡
1/KJ461874	Bacterial	5.2 × 10^3^	21, M	Bacteria	0	2012 Dec 4	VP2, 2786–4495, 0.88
2/KJ461875	Bacterial	1.9 ×10^4^	38, M	Bacteria	0	2013 Jan 6	VP2, 2786–4495, 0.71
3/§	Bacterial	1.9 × 10^3^	53, M	Bacteria	0	2013 Jan 11	§
4/KJ461876	Bacterial	3.7 × 10^3^	46, M	Bacteria	0	2013 Apr 27	VP2, 2786–4495, 0.76
5/KJ461877	Viral	3.4 × 10^3^	77, M	Norovirus	0	2013 Apr 19	VP2, 2786–4495, 1.60
6/KJ461878¶	Viral	3.6 × 10^3^	89, F	Norovirus	0	2013 Apr 20	Partial NS, 16–1080, 1.13
7/KJ461878¶	Viral	3.2 × 10^4^	89, F	Norovirus	Norovirus	2013 Apr 23	VP2, 2786–4495, 1.36

*Pt. ID, patient identification; VP2, viral protein 2; NS, nonstructural. †Samples originally sent to HUSLAB (Helsinki, Finland) for bacterial diagnosis were analyzed for *Salmonella* spp*., Shigella* spp*., Campylobacter* spp*., Yersinia* spp*., Vibrio cholerae*, and *Escherichia coli* (subtypes enterohemorraghica, enteropatogena, enterotoxigenic and enteroagregativa) by using culture or PCR The bufavirus sequences were submitted to GenBank (accession nos. KJ461874-KJ461879); bufavirus-positive samples could not be analyzed for the presence of pathogens other than those originally tested for because the samples had been discarded.  ‡ Sequence divergence analyzed by using the DNA distance matrix in BioEdit (http://www.mbio.ncsu.edu/BioEdit/bioedit.html). §This sample was positive for bufavirus by quantitative PCR. However, we were not able to amplify another region of the virus from this sample, likely due to a low amount of the virus in the sample, which had the lowest copy number among the positive samples. As a result, no patient identification number was assigned. ¶Samples from the same patient, collected 4 days apart.

Seven fecal samples collected from adults in Finland contained BuV DNA, indicating that circulation of the virus is restricted neither to children nor to Africa. However, the low DNA loads in all the positive samples suggest that BuV might not be the primary cause of these cases of gastroenteritis. A known gastroenteritis-inducing pathogen (norovirus) was found in 1 of the 7 BuV-positive samples. We did not observe any clustering of the 7 positive samples into a specific period (Table).

Although the association with gastroenteritis seems weak, BuV might cause symptoms of other types. We did not include feces from healthy subjects for comparison. The identified BuV DNA in our samples could originate from previous or current infections unrelated to gastroenteritis, or be associated with prolonged virus secretion in the respiratory or digestive tracts, a phenomenon shown, e.g., for (*9**,**10*). Acquisition of the virus from a food source cannot be ruled out, although 1 patient harbored the DNA for at least 4 days, during which a 10-fold increase in viral load was observed.

Overall, this study shows that BuV circulates in northern Europe and can be found in the feces of patients with gastroenteritis. Despite the absence of known pathogens among 6 of 7 BuVs-shedding patients, the causative role of BuV in gastroenteritis remains uncertain. Serologic studies will help clarify a possible association between BuVs and diarrhea or other diseases.

Technical Appendix Bufavirus quantitative PCR and phylogenetic analylsis.

## References

[1] Allander T, Tammi MT, Eriksson M, Bjerkner A, Tiveljung-Lindell A, Andersson B. Cloning of a human parvovirus by molecular screening of respiratory tract samples. Proc Natl Acad Sci U S A. 2005;102:12891–6 . 10.1073/pnas.0504666102 16118271 PMC1200281

[2] Jones MS, Kapoor A, Lukashov VV, Simmonds P, Hecht F, Delwart E. New DNA viruses identified in patients with acute viral infection syndrome. J Virol. 2005;79:8230–6 . 10.1128/JVI.79.13.8230-8236.2005 15956568 PMC1143717

[3] Arthur JL, Higgins GD, Davidson GP, Givney RC, Ratcliff RM. A novel bocavirus associated with acute gastroenteritis in Australian children. PLoS Pathog. 2009;5:e1000391 . 10.1371/journal.ppat.1000391 19381259 PMC2663820

[4] Kapoor A, Slikas E, Simmonds P, Chieochansin T, Naeem A, Shaukat S, A newly identified bocavirus species in human stool. J Infect Dis. 2009;199:196–200 . 10.1086/595831 19072716 PMC2678954

[5] Kapoor A, Simmonds P, Slikas E, Li L, Bodhidatta L, Sethabutr O, Human bocaviruses are highly diverse, dispersed, recombination prone, and prevalent in enteric infections. J Infect Dis. 2010;201:1633–43 . 10.1086/652416 20415538 PMC2902747

[6] Phan TG, Vo NP, Bonkoungou IJ, Kapoor A, Barro N, O'Ryan M, Acute diarrhea in West African children: diverse enteric viruses and a novel parvovirus genus. J Virol. 2012;86:11024–30 . 10.1128/JVI.01427-12 22855485 PMC3457132

[7] Cotmore SF, Agbandje-McKenna M, Chiorini JA, Mukha DV, Pintel DJ, Qiu J, The family *Parvoviridae.* Arch Virol. 2013;•••: Epub ahead of print . 10.1007/s00705-013-1914-1 24212889 PMC4013247

[R8] 8. Antikainen J, Kantele A, Pakkanen SH, Laaveri T, Riutta J, Vaara M, et al. A quantitative polymerase chain reaction assay for rapid detection of 9 pathogens directly from stools of travelers with diarrhea. Clin Gastroenterol Hepatol. 2013;11:1300,1307.e3. PubMed 10.1016/j.cgh.2013.03.037 23639597

[9] Martin ET, Fairchok MP, Kuypers J, Magaret A, Zerr DM, Wald A, Frequent and prolonged shedding of bocavirus in young children attending daycare. J Infect Dis. 2010;201:1625–32 . 10.1086/652405 20415535 PMC2862123

[10] Kapusinszky B, Minor P, Delwart E. Nearly constant shedding of diverse enteric viruses by two healthy infants. J Clin Microbiol. 2012;50:3427–34 . 10.1128/JCM.01589-12 22875894 PMC3486243

