# Supplementary material for: Bufavirus in Feces of Patients with Gastroenteritis, Finland
Source: Emerg Infect Dis. 2014 Jun;20(6):1077–9. doi: 10.3201/eid2006.131674 (PMC4036783; doi:10.3201/eid2006.131674)
Supplement: Technical Appendix — Bufavirus quantitative PCR and phylogenetic analylsis. [file 13-1674-Techapp-s1.pdf]

Article DOI: 10.3201/eid.2006.131674

Bufavirus in Feces of Patients with Gastroenteritis, Finland

Technical Appendix

Bufavirus Quantitative PCR

A hydrolysis-probe based quantitative PCR (qPCR) was designed on the conserved NS1 region of the bufavirus genome, based on the bufavirus sequences published in GenBank (accessed 1st Oct 2012). The following primers and probe were selected (nucleotides corresponding to JX027295): BuV fwd (705-730nt), 5′-ACA GTG TAG ACA GTG GAT TCA AAC TT-3′; BuV rev (830-806nt), 5′-GTT GTG GTT GGA TTG TGG TTA GTT C-3′; BuV qPCR probe (789-762nt), 5′-FAM-CGG AAG AGA TTT TGA CAG TGC YTA GCA A-BHQ1-3′. The qPCR reaction consisted of 1x Maxima Probe qPCR Master Mix (Fermentas, Vilnius, Lithuania), 30 nM of ROX passive reference dye, 0.5 µM of each primer, 0.2 µM of BuV qPCR probe, 5 µl of template and H_2O up to a final volume of 25 µl. DNA was amplified by the Stratagene Mx3005P machine with the following program: initial denaturation of 10 min at 95°C followed by 45 cycles of 15 s at 95°C and 1 min at 60°C.

For controls in methods set up and optimization, a 3.5kb piece of BuV1 (16-3577 nt, JX027295) and 830 nt of BuV2 (1-829 nt, JX027297) were amplified from the original fecal supernatants and cloned into the pSTBlue vector *(6)*. These plasmids were used in testing of sensitivity and specificity of the qPCR with and without 500 ng human DNA per reaction (HEK293 cell line DNA) and extracted DNA from fecal samples. Specificity of the assay was tested with other human parvoviruses, human parvovirus B19 (B19V), HBoV1, and PARV4. A 10-fold dilution series of the BuV1 plasmid was used in the generation of a standard curve for quantification.

The analytical sensitivity of the RT-qPCR assay was 5-10 copies per reaction for both of the BuV plasmids, and the assay showed linearity over a range of 50 to $\geq 5x10^6$ copies per reaction. Human DNA or fecal DNA extracts did not interfere with the amplification or cause

false positives. Furthermore, the qPCR did not amplify B19V, HBoV1 or PARV4 DNAs even when using very high copy-number templates. All negative controls, i.e. water, human DNA and negative fecal samples, remained negative in all assays.

DNA Extractions from Fecal Samples

The DNA from the fecal samples in the bacterial cohort was extracted from 900 µl of 10% fecal suspension with an automated Tecan pipetting robot system (Männedorf, Switzerland) following the NucliSens EasyMag nucleic acid extraction protocol (bioMerieux, Marcy l'Etoile, France). The nucleic acids in the viral cohort were extracted from 200 µl of a 10% fecal suspension in Tris-NaCl-$CaCl_2$ buffer with the MagnaPure LC Total nucleic acid isolation kit (Roche Applied Sciences, Mannheim, Germany).

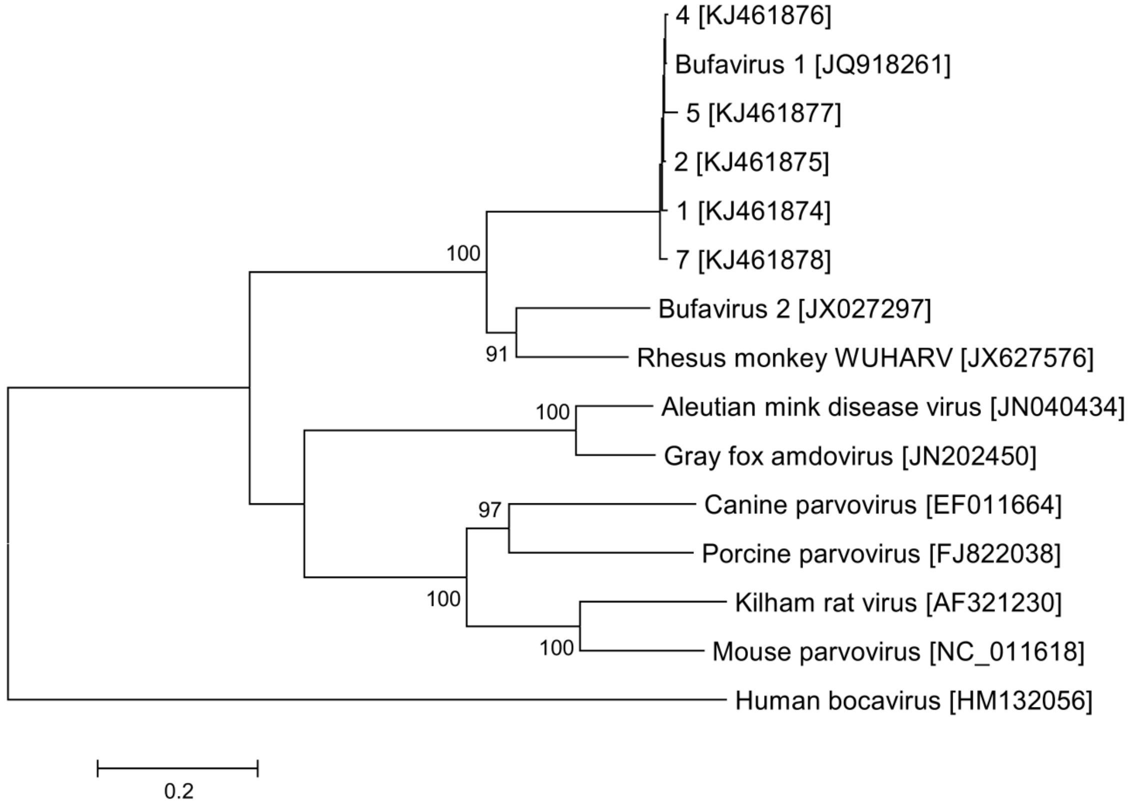

Technical Appendix Figure

Phylogenetic analysis of the viral protein 2 of bufavirus strains in Finland and related parvoviruses. Genetic distances were calculated by the Kimura 2-parameter method (PHYLIP), and a phylogenetic tree with 100-bootstrap resampling of the alignment data sets was generated by using the neighbor-joining method. Scale bar indicates the number of amino acid substitutions per position.
